# Analyzing Citizens’ and Health Care Professionals’ Searches for Smell/Taste Disorders and Coronavirus in Finland During the COVID-19 Pandemic: Infodemiological Approach Using Database Logs

**DOI:** 10.2196/31961

**Published:** 2021-12-07

**Authors:** Milla Mukka, Samuli Pesälä, Charlotte Hammer, Pekka Mustonen, Vesa Jormanainen, Hanna Pelttari, Minna Kaila, Otto Helve

**Affiliations:** 1 University of Helsinki Helsinki Finland; 2 Epidemiological Operations Unit City of Helsinki Helsinki Finland; 3 European Programme for Intervention Epidemiology Training Solna Sweden; 4 Department of Health Security Finnish Institute for Health and Welfare Helsinki Finland; 5 Duodecim Medical Publications Ltd Helsinki Finland; 6 Department of Public Health University of Helsinki Helsinki Finland; 7 Performance Assessment of the Health and Social Service System Finnish Institute for Health and Welfare Helsinki Finland; 8 Clinicum University of Helsinki Helsinki Finland; 9 Pediatric Research Center Children's Hospital Helsinki University Hospital, University of Helsinki Helsinki Finland

**Keywords:** COVID-19, SARS-CoV-2, smell disorders, taste disorders, information-seeking behavior, health personnel, statistical models, medical informatics

## Abstract

**Background:**

The COVID-19 pandemic has prevailed over a year, and log and register data on coronavirus have been utilized to establish models for detecting the pandemic. However, many sources contain unreliable health information on COVID-19 and its symptoms, and platforms cannot characterize the users performing searches. Prior studies have assessed symptom searches from general search engines (Google/Google Trends). Little is known about how modeling log data on smell/taste disorders and coronavirus from the dedicated internet databases used by citizens and health care professionals (HCPs) could enhance disease surveillance. Our material and method provide a novel approach to analyze web-based information seeking to detect infectious disease outbreaks.

**Objective:**

The aim of this study was (1) to assess whether citizens’ and professionals’ searches for smell/taste disorders and coronavirus relate to epidemiological data on COVID-19 cases, and (2) to test our negative binomial regression modeling (ie, whether the inclusion of the case count could improve the model).

**Methods:**

We collected weekly log data on searches related to COVID-19 (smell/taste disorders, coronavirus) between December 30, 2019, and November 30, 2020 (49 weeks). Two major medical internet databases in Finland were used: Health Library (HL), a free portal aimed at citizens, and Physician’s Database (PD), a database widely used among HCPs. Log data from databases were combined with register data on the numbers of COVID-19 cases reported in the Finnish National Infectious Diseases Register. We used negative binomial regression modeling to assess whether the case numbers could explain some of the dynamics of searches when plotting database logs.

**Results:**

We found that coronavirus searches drastically increased in HL (0 to 744,113) and PD (4 to 5375) prior to the first wave of COVID-19 cases between December 2019 and March 2020. Searches for smell disorders in HL doubled from the end of December 2019 to the end of March 2020 (2148 to 4195), and searches for taste disorders in HL increased from mid-May to the end of November (0 to 1980). Case numbers were significantly associated with smell disorders (*P*<.001) and taste disorders (*P*<.001) in HL, and with coronavirus searches (*P*<.001) in PD. We could not identify any other associations between case numbers and searches in either database.

**Conclusions:**

Novel infodemiological approaches could be used in analyzing database logs. Modeling log data from web-based sources was seen to improve the model only occasionally. However, search behaviors among citizens and professionals could be used as a supplementary source of information for infectious disease surveillance. Further research is needed to apply statistical models to log data of the dedicated medical databases.

## Introduction

COVID-19 is a contagious respiratory illness caused by the novel coronavirus (SARS-CoV-2). It has been prevailing worldwide since the beginning of 2020 [1]. Various symptoms may be related to COVID-19, such as smell and taste disorders [2]. Loss of smell was a new COVID-19 symptom first reported by the British Rhinology Society in March 2020 [3], and a high prevalence of smell and taste dysfunction among patients with COVID-19 has been found [2]. Internet users seek information on COVID-19 during the pandemic. Infodemiology is an area of science research that scans the internet for user-contributed health-related content [4], with the goal of improving public health [5]. It offers digital data (internet, social media) that can be collected and analyzed in real time, to understand how and why people search for health information and how it affects the data [5]. In terms of disease surveillance, search queries on health-related information may serve as early predictors of population health compared with traditional epidemiology [6]. Besides, the combination of internet surveillance data (online searches) and traditional surveillance data (such as laboratories and physicians’ diagnoses) has been shown to provide additional information for alerting and informing the public, as well as better targeting public health policies [7]. However, predicting the course of the pandemic may be difficult due to a variety of factors that have been found to contribute to an infectious disease outbreak [5,8]. Previously, data from search engines have generated high hopes for contributing to outbreak surveillance [7,9-11]. For example, for influenza outbreaks, Google Trends and Google Flu Trends have been used as models for predicting incidences of the disease [10,11]. In China, an increase in internet searches on coronavirus was observed 5-10 days before the disease outbreak and was found to predict an increase in suspected and laboratory-confirmed COVID-19 cases [8]. Strong positive correlations were also found between initial symptoms of COVID-19 and Google search interests [12]. Refining the data signal by reducing surrounding noise remains a big challenge in the field of infodemiology [6]. One of the problems is that general search engines and other internet platforms cannot characterize the users performing the searches, including both citizens and health care professionals (HCPs). Citizens’ searches may be more easily influenced by epidemiologically irrelevant factors such as publicity. However, previous studies have shown that focusing research on the dedicated databases used by HCPs provides reliable data for the surveillance of infectious diseases [13,14].

Infodemiology has been acknowledged by public health organizations and the World Health Organization (WHO) as an important emerging scientific field of practice during the pandemic [15], and plays an important role in health informatics research [4]. Infodemiology can be applied to web-based sources of COVID-19–related smell and taste disorders. Many studies on COVID-19–related searches for loss of smell and taste have analyzed information seeking from Google and Google Trends [16-18]. Strong correlations have been found between COVID-19 cases and Google searches on loss of smell and coronavirus information in several countries [16,18]. However, some studies have shown no correlation between Google searches for loss of smell/taste and COVID-19, and their usability as a web-based surveillance method has also been criticized [17]. Novel infodemiological approaches are needed to analyze searches for COVID-19 symptoms from internet databases. Little data exist on how statistical modeling of website log data on smell/taste disorders and coronavirus from the dedicated evidence-based medicine (EBM) sources used by citizens and professionals could enhance disease surveillance and outbreak detection during the COVID-19 pandemic.

In Finland, Health Library (HL) is by far the most frequently used general public health portal on the internet. More than 50 million articles are opened per year by a population of 5.3 million in Finland. The service is free of charge with no advertising, and is provided by the Finnish Medical Society Duodecim, which is a 140-year-old scientific organization with over 24,000 physician members (>90% of the Finnish physicians). Physician’s Database (PD), an online source, provides medical information for HCPs. The articles in HL have been written and updated by the same physicians that are authors of the PD aimed at HCPs. Recommendations and evidence summaries are published in PD and are referenced in HL articles for the general public. Most of the HL articles are accessed via Google searches by the general public, with more than 80% using mobile devices. The remaining users (approximately 20%) access the service directly via the web address [19]. When producing the medical articles (>10,000 in total) in HL, the quality criteria of Health On the Net [20] are met.

Previous studies [13,14] on these databases have showed that HCPs’ searches in PD on Lyme borreliosis and influenza precede the trends shown by current outbreak surveillance data (public primary care diagnoses and laboratory findings). Therefore, we hypothesized that PD searches could be used as a supplementary source of information for examining COVID-19 spread. In addition, citizens’ searching behavior of web-based health information during epidemics may closely follow those of HCPs [21]. Therefore, we also hypothesized that HL information seeking among citizens could be used as a supplementary source of information for disease surveillance [21]. Lyme borreliosis and influenza show seasonal patterns of cases and searches [13,14,21], while COVID-19 may not show seasonal patterns so clearly. Of note, the log data on COVID-19 in HL and PD have previously not been analyzed using statistical models. Here we hypothesized that our models would provide a novel approach to analyze web-based seeking behaviors among citizens and professionals. The aim of this study was (1) to assess whether citizens’ and HCPs’ searches for smell disorders, taste disorders, and coronavirus relate to epidemiological data on COVID-19, and (2) to test our negative binomial regression modeling (ie, whether the inclusion of the case count could improve the model).

## Methods

### Databases and Register

We collected weekly coronavirus log data from HL and PD (December 30, 2019 to November 30, 2020) in Finland [22]. We used the number of searches (ie, article links opened by clicking on the entries within the database) from the 3 HL articles (“Smell disorders,” “Taste disorders,” and “New Coronavirus”) and PD articles (“Smell disorders,” “Taste disorders,” and “Coronavirus”). The taste disorder article was published on the HL platform in mid-May 2020. These database logs were combined with the register data on the number of COVID-19–positive test results (cases) from the Finnish National Infectious Diseases Register of the Finnish Institute for Health and Welfare [23]. The time scale (49 weeks) included the first and second waves of COVID-19 cases. The first wave started in Week 5 (first COVID-19 case) but a clear increase in cases appeared in Week 11, and the wave ended in Week 26. The second wave occurred between Weeks 27 and 49.

### Descriptive Statistical Analysis

First, we plotted the searches for all 3 indicators (smell disorders, taste disorders, and coronavirus) over the last 10 years (2010-2020) in both databases (HL and PD) on a weekly basis to see if there were any visual trends in patterns. Second, we assessed weekly searches for the 3 indicators in HL and PD, as well as COVID-19 cases during December 30, 2019, and November 30, 2020 (49 weeks), to compare if they preceded or appeared at the same time (peaked) in patterns.

### Time-Series Analysis

Third, we ran time-series analyses using negative binomial regression models of the number of searches explained by time (week) and weekly cases of COVID-19. For each model, we determined if the case count was a significant predictor. We assumed statistical significance at *P*<.05. We also performed likelihood ratio tests (LRTs) using analysis of variance (ANOVA) and Akaike information criterion (AIC) to assess model fit. Time-series analyses were conducted in R (R version 4.0.5; packages “zoo” and “MASS”) using RStudio (R Foundation for Statistical Computing) [24]. Log data were analyzed anonymously using internet protocol addresses of the purchasers of the PD, not the personal internet protocol addresses of the professionals. Thus, no individual HCP performing the searches can be identified. No ethical statements were needed.

## Results

### Descriptive Statistical Analysis

**Figure 1:**
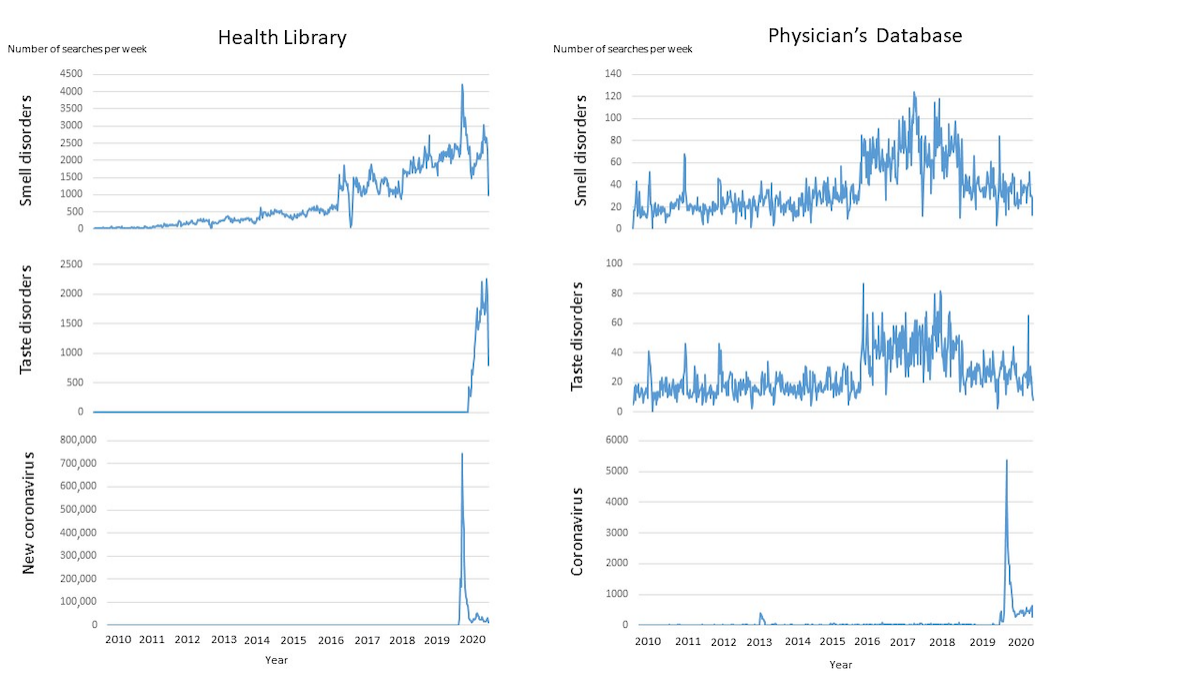
Health Library and Physician’s Database weekly searches for smell disorders, taste disorders, and coronavirus in Finland during 2010-2020.

**Figure 2:**
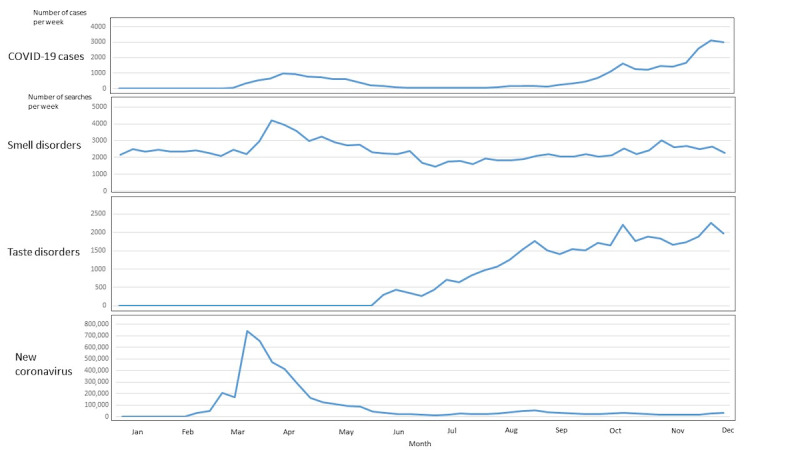
COVID-19 cases and Health Library searches for smell disorders, taste disorders, and new coronavirus in Finland between December 30, 2019, and November 30, 2020.

**Figure 3:**
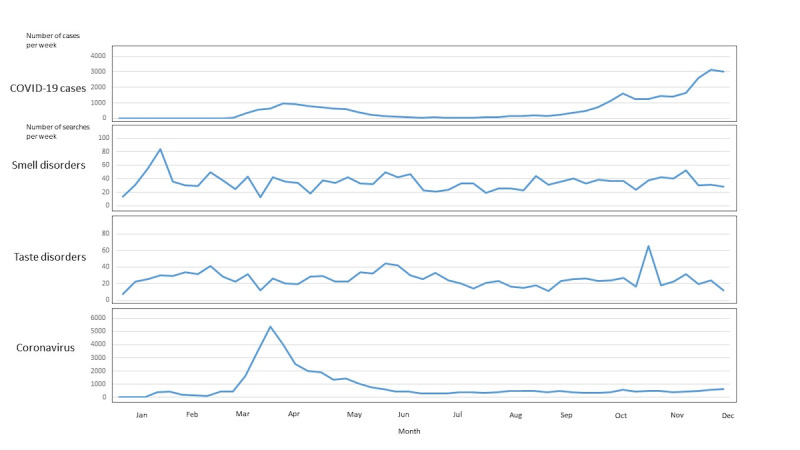
COVID-19 cases and Physician’s Database searches for smell disorders, taste disorders, and coronavirus in Finland between December 30, 2019, and November 30, 2020.

When plotting the searches for smell disorders, taste disorders, and coronavirus over the last 10 years (2010-2020) in HL and PD, we found that time lag was unlikely upon visual inspection, seasonality was not assessable due to COVID-19 waves, and nothing indicative appeared in pre-2020 data (Figure 1). Between December 30, 2019, and November 30, 2020 (Figures 2 and 3), coronavirus searches drastically increased in HL (from 0 to 744,113 in Weeks 1-11) and PD (from 4 to 5375 in Weeks 1-13), prior to the first wave of COVID-19 cases (starting in Week 11). Citizens’ searches for smell disorders in HL doubled (from 2148 to 4195) from the end of December 2019 (Week 1) to the end of March 2020 (Week 13). Citizens’ searches for taste disorders in HL increased (from 0 to 1980) from mid-May (Week 21) to the end of November 2020 (Week 49). Professionals’ searches for smell and taste disorders in PD showed uneven patterns (Figure 3). The maximum and minimum months and weeks, as well as the total number of searches and cases are shown in Table 1.

**Table 1:** The maximum and minimum months and weeks of searches and cases, and the total number of Health Library and Physician’s Database searches for smell disorders, taste disorders, and coronavirus, as well as COVID-19 cases in Finland between December 30, 2019, and November 30, 2020.

Database		Maximum number of searches or cases (peaks)			Minimum number of searches or cases			Total number of searches or cases (cumulative)
		Month (week)	Searches in maximum week	Month (week)		Searches in minimum week		
**Health Library**								
	Searches for smell disorders	March (13)	4195	June (26)		1468	117,477	
	Searches for taste disorders	November (48)	2262	December to May (1-21)		0	37,114	
	Searches for new coronavirus	March (11)	744,113	December to February (1-6)		0	4,395,898	
**Physician’s Database**								
	Searches for smell disorders	January (4)	84	March (12)		13	1706	
	Searches for taste disorders	October (43)	65	December (1)		7	1235	
	Searches for coronavirus	March (13)	5375	December (1)		4	39,779	
COVID-19 cases		November (48)	3134	December to February (1-4)		0	28,385	

### Time-Series Analysis

**Figure 4:**
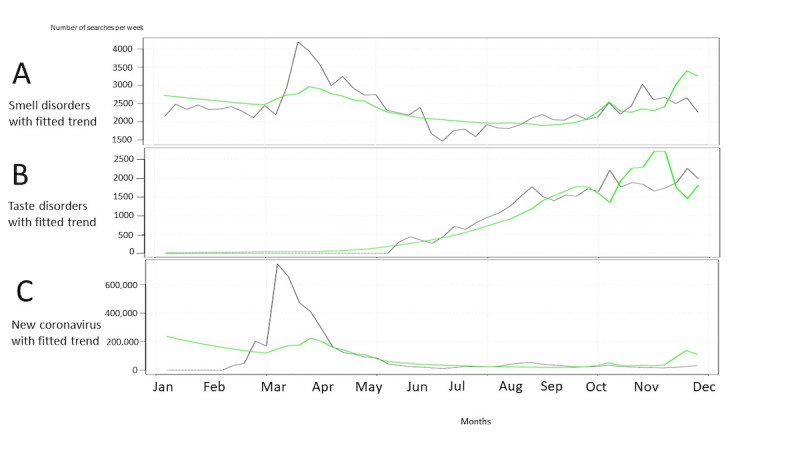
Health Library weekly searches (gray line) with fitted trends (green line) for smell disorders (A), taste disorders (B), and new coronavirus (C) in Finland between December 30, 2019, and November 30, 2020. Fitted trends took into account time and COVID-19 cases.

**Figure 5:**
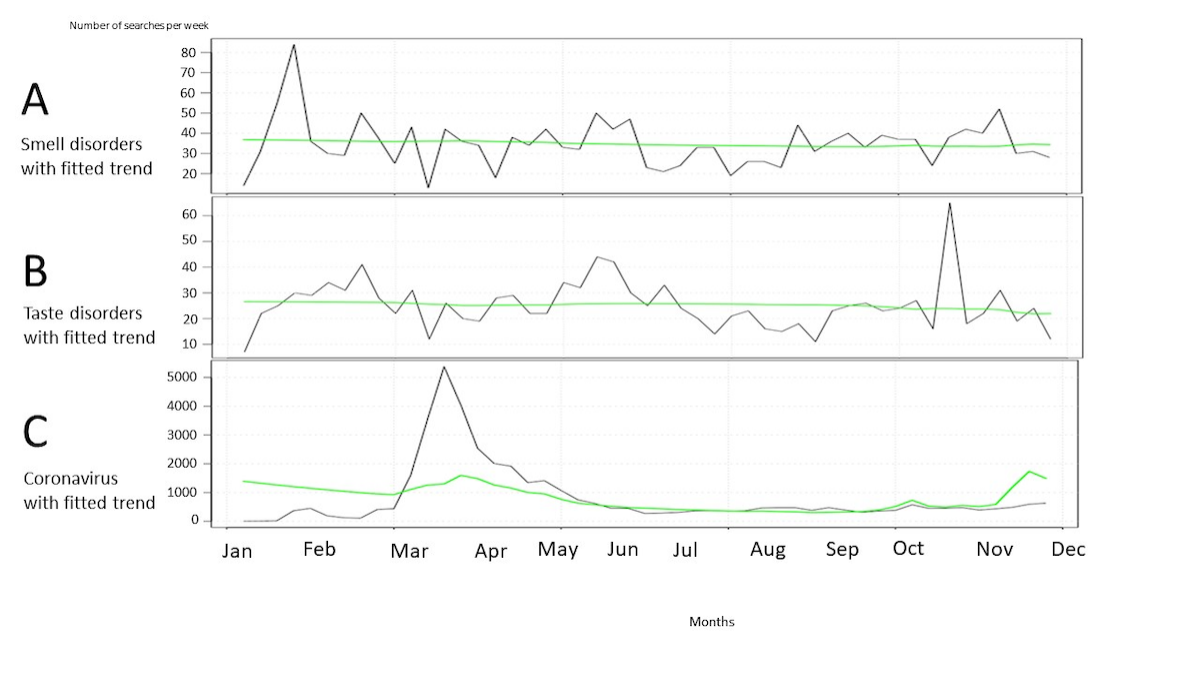
Physician Database weekly searches (gray line) with fitted trends (green line) for smell disorders (A), taste disorders (B), and coronavirus (C) in Finland between December 30, 2019, and November 30, 2020. Fitted trends took into account time and COVID-19 cases.

Smell disorder searches in HL were significantly associated with case numbers in the time-series analysis (*P*<.001; Figure 4A). Including the case numbers in the model of smell disorders did significantly improve the model (LRT ANOVA *P*<.001, AIC reduced from 752.71 to 725.58). While case numbers were associated with taste disorders in HL (*P*<.001), the model was statistically significant improved (LRT ANOVA *P*<.001, AIC reduced from 10,464.04 to 5524.93) but not performing adequately based on visualization (Figure 4B). Even after including case numbers and new coronavirus searches in HL, the model did not improve (LRT ANOVA *P*>.99, AIC increased from 1141.26 to 5,642,226.89; Figure 4C). When plotting PD searches for coronavirus and COVID-19 case numbers, the model improved (LRT ANOVA *P*=.001, AIC reduced from 754.74 to 745.94; Figure 5C). For smell and taste disorders, there was no improvement in the model (Figure 5A and Figure 5B). The results of LRT ANOVA and AIC are presented in Table 2.

**Table 2:** Health Library and Physician’s Database searches for smell disorders, taste disorders, and coronavirus fitted with a trend of COVID-19 cases, including *P* values of cases in model, LRT^a^, ANOVA^b^, and AIC^c^, and model improvement information.

Database		*P* value of cases in model	LRT ANOVA, *P* value	AIC	Model improvement
**Health Library**					
	Searches for smell disorders	<.001	<.001	From 752.71 to 725.58 (Reduced)	Improved
	Searches for taste disorders	<.001	<.001	From 10,464.04 to 5524.93 (Reduced)	Improved
	Searches for new coronavirus	<.001	>.99	From 1141.26 to 5,642,226.89 (Increased)	Not improved
**Physician’s Database**					
	Searches for smell disorders	.76	.77	From 380.26 to 382.17 (Increased)	Not improved
	Searches for taste disorders	>.99	.63	From 358.68 to 360.46 (Increased)	Not improved
	Searches for coronavirus	<.001	.001	From 754.74 to 745.94 (Reduced)	Improved

^a^LRT: likelihood ratio test.

^b^ANOVA: analysis of variance.

^c^AIC: Akaike information criterion.

## Discussion

### Principal Findings

In our study, we were able to characterize the searching behaviors of citizens and HCPs during the COVID-19 epidemic in Finland 2020. We found that information seeking on coronavirus preceded the cases in the first wave, but not in the second one. Searches for smell and taste disorders showed a visually clear pattern in citizens’ searching, while HCPs’ searches remained uneven throughout the months. A clear model improvement was found when fitting the model of case numbers and plotting citizens’ smell and taste disorder searches and professionals’ coronavirus searches, respectively.

### Citizens’ HL Searches

For smell disorders, when we plotted HL searches and fitted a model that took into account time and COVID-19 case numbers in Finland, the case numbers could explain some of the dynamics of the search. This means that the model has improved from a statistical point of view. However, a visual pattern was not performing well. For taste disorders, when we plotted HL searches and fitted a model that took into account time and COVID-19 cases, the case numbers could again explain the dynamics of searches. This indicated that compared with a model that would only include time, there is an improvement when the COVID-19 incidence is added to the model. However, this model was also not performing very well visually, showing deviation at the end of the year. When plotting new coronavirus searches in HL and fitting a model taking into account time and COVID-19 cases, the case numbers could not explain the dynamics of the search. This means that inclusion of COVID-19 cases does not improve the model statistically. New coronavirus searches and COVID-19 cases seemed to coincide in this model, indicating that with more cases, more users read up on coronavirus and associated symptoms from internet sources. However, it could also mean that for the smell and taste disorders, more people get these symptoms because of COVID-19, and therefore look them up from internet sources. It is not possible to determine this interpretation from the data, but as the searches for coronavirus follow the same pattern, the plausible explanation is that citizens seek web-based information on new coronavirus and its associated symptoms (smell and taste disorders).

### Professionals’ PD Searches

When plotting PD searches for coronavirus and fitting a model that took into account time and COVID-19 case numbers in Finland, the case numbers could explain some of the dynamics of the search. These results show that the model has improved. However, improvement appears only when the cases are up and professionals search more for information. For smell and taste disorders, there is no improvement in the models, possibly because there were seen simply as symptoms of COVID-19, thus not warranting investigation as individual disorders. In PD searches for smell and taste disorders, a temporary increase was seen in patterns during 2017-2019, caused by changes in logs on the platform. However, it did not affect our results. Our findings showed that modeling professionals’ seeking behavior on COVID-19 did not perform as well as we had hypothesized.

### Comparison With Prior Work

Prior studies have assessed the searches for coronavirus and smell/taste disorders related to COVID-19 from Google and Google Trends [16-18]. The usability of web-based surveillance methods has also been criticized [17]. In infodemiology, refining the data signal by reducing surrounding noise remains a big challenge [6]. General search engines and the results they provide may yield unreliable health information for HCPs and citizens, and engines cannot distinguish the users, possibly resulting in poorer detection of infectious diseases based on internet searches. In Finland, PD is aimed at HCPs, thus we are able to assess the searches performed by HCPs. Prior studies have found that PD searches for Lyme borreliosis [13] and influenza [14] preceded the trends shown by current outbreak surveillance data (public primary care diagnoses and laboratory findings). We concluded that PD searches could be used as a supplementary source of information for disease surveillance. Besides, a prior study [21] has found that citizens’ searches in HL followed epidemiological data on Lyme borreliosis. Both HL and PD are built upon EBM sources. General search engines may yield unreliable results that will lead searchers to the online sources of misinformation on COVID-19 [25]. Our study has demonstrated the difference between citizens’ and HCPs’ database search behaviors on coronavirus, as well as smell and taste disorders and their relation to COVID-19 cases with statistical model testing during the COVID-19 epidemic in Finland 2020. Our results strengthen prior findings of using the searches in HL and PD as a supplementary source of information for infectious disease surveillance.

### Strengths and Limitations

The strengths of our study were representativeness (HCPs using PD) and timeliness (real-time internet databases), as well as reliable medical internet sources (citizens and HCPs using EBM databases). Some limitations of this study should be taken into consideration. Coronavirus searches in HL and PD began to increase prior to the outbreak and continued rising during the first wave, and then decreased. However, the increasing pattern was not seen during the second wave. A possible reason may be that the second wave appeared very soon after the first one, thus making the disease more familiar to HCPs who needed less information on the virus. In addition, daily news and media publications on COVID-19 may have had a huge impact on both citizens’ and HCPs’ information seeking behavior on the internet and from web-based sources. Initial curiosity in the novel disease resulted in an increased searching pattern. However, citizens and HCPs may have been later fatigued by overwhelming media coverage of COVID-19 or they went to other sources, resulting in a rapid decrease in searches, although confirmed cases remained high during the course of the pandemic. Patients with COVID-19 may be asymptomatic or presymptomatic [26], thus they may not be eager to seek internet information on COVID-19. This may also decrease searches in the databases. A previous study [17] has suggested that the decrease in Google searches for taste and smell loss after the first months of the pandemic can be explained by news on digital media. Besides, genuine interest on self-symptoms before they become broadly known to the general public may have faded [17]. Prior studies have found that Google searches for smell and taste loss varied between countries [27] but remained at a higher level after the beginning of the pandemic [27,28]. In our study, searches for smell and taste disorders showed visually better prediction among citizens than HCPs, possibly indicating that loss of smell or taste may have been the only concerning symptom of mild COVID-19 cases. Therefore, citizens may have searched for information from web-based sources about these symptoms rather than visiting a doctor. We also found that HCPs’ PD searches for smell and taste disorders showed no improvement in models due to the small number of searches. There were no citizens’ HL searches for taste disorders from the end of December 2019 to mid-May 2020, since the first article was published in mid-May and searching started to increase. We cannot distinguish if the increase in searches resulted from citizens’ interest in a novel article or in COVID-19–related symptoms. However, we assume the increase may include both.

### Conclusions

Our study has visually shown how much and how fast citizens and HCPs began to seek health information from web-based sources at the start of the COVID-19 outbreak and how this searching has carried on during the pandemic in Finland 2020. Modeling log data statistically improved the model only occasionally. However, citizens’ and professionals’ search behaviors could be used as an additional source of information for infectious disease surveillance. Further research is needed to apply statistical models to log and register data of the dedicated reliable medical sources, as well as to assess predictive values of smell and taste disorder searches on the internet. Novel infodemiological approaches provide an understanding of citizens’ and professionals’ information seeking behaviors on COVID-19 from web-based databases. Results could be used in decision making, planning, and research, in collaboration with experts working in various fields of public health medicine and health informatics.

## Abbreviations

AIC: Akaike information criterion
ANOVA: analysis of variance
EBM: evidence-based medicine
HCPs: health care professionals
HL: Health Library
LRT: likelihood ratio test
PD: Physician’s Database
